# Acceptability and feasibility of a group intervention for long COVID in Johannesburg, South Africa: a mixed-method study

**DOI:** 10.3389/frhs.2025.1666387

**Published:** 2025-10-30

**Authors:** Rupa Ramachandran, Farzana Sathar, Pride Mokome, Nkululeko Mathabela, Ency Mahlase, Salome Charalambous, Andrea Rachow, Nicole Audrey Glover, Olena Ivanova

**Affiliations:** ^1^ Deggendorf Institute of Technology, European Campus Rottal-Inn, Pfarrkirchen, Germany; ^2^ Implementation Research Division, The Aurum Institute, Johannesburg, South Africa; ^3^ Institute of Infectious Diseases and Tropical Medicine, LMU University Hospital, LMU Munich, Munich, Germany; ^4^ German Centre for Infection Research (DZIF), Partner Site Munich, Munich, Germany; ^5^ Unit Global Health, Helmholtz Zentrum München, German Research Centre for Environmental Health (HMGU), Neuherberg, Germany

**Keywords:** long COVID, South Africa, acceptability, feasibility, group care

## Abstract

**Background:**

COVID-19 affected 777 million people globally, with 7.1 million deaths. In Africa, 9.6 million cases and 176,000 deaths were reported. Long COVID, a significant consequence of the COVID-19, presented by chronic symptoms, affects the physical and mental health, thereby impacting the quality of life. While high-income countries implemented rehabilitation programs for managing long COVID symptoms, low- and middle-income countries faced healthcare disparities. In South Africa, limited multidisciplinary interventions were evident. This study aimed to assess the acceptability and feasibility of an 8-week rehabilitation and self-management program for long COVID using mixed-methods approach in Johannesburg.

**Methods:**

Patients and hospital staff who suffered from at least one symptom of long COVID for a period of two months and who consented to participate in the intervention were recruited from Tembisa Provincial Tertiary Hospital. The recruitment was from July to October 2023. Questionnaires were administered and interviews with selected participants were conducted to assess the acceptability and feasibility of the intervention. A descriptive analysis was carried out for the quantitative data, and a deductive thematic analysis was used for the interviews.

**Results:**

The participants had positive perceptions towards the design of the intervention, delivery, materials used and support by research staff and external consultants such as dietitians, physiotherapists, and psychologists. The participants stated that the intervention had improved their knowledge of long COVID and increased their self-confidence. Major barriers related to the intervention perceived by the participants were infrastructure, time and language. Recommendations from the participants included expanding the intervention at the community level and extending the duration of the intervention beyond 8-weeks.

**Conclusion:**

This pilot intervention, that aimed to manage the symptoms of long COVID, was well accepted by the participants and achieved its intended outcome. Similar interventions are required at the clinical as well as community levels.

## Introduction

COVID-19, a highly transmissible acute respiratory infection, affected 777 million people and caused 7.1 million deaths worldwide (1, 2). In the WHO African region, 9.6 million cases and 176,000 deaths have been reported (1, 2). The pandemic also had profound social as well as economic impacts on the society (3). The social distancing, cancellation of social events, shutting down of enterprises and businesses and border closures affecting tourism and rise in food insecurity due to market disruptions contributed to significant hardships and challenges (3). A significant and lasting consequence of COVID-19 is long COVID or post-acute sequelae of COVID-19 (PASC). According to WHO, long COVID is defined as “*continuation or development of new symptoms 3 months after the initial SARS-CoV-2 infection, with these symptoms lasting for at least 2 months with no other explanation”* (4). It is considered that 10%–20% of people infected by SARS-CoV-2 can have chronic symptoms that can be diagnosed as long COVID (4). Chen et al. published the global pooled prevalence of long COVID to be 0.43 (95% CI: 0.39, 0.46 (5). Almost 20 symptoms were linked to long COVID (4), with fatigue, cognitive dysfunction, dyspnoea, sleep problems and joint pain being the most prevalent symptoms (5).

The impacts of long COVID in low- and middle-income countries (LMICs), especially in Africa, has been understudied. A meta-analysis study in Africa found that nearly 50% of the patients with previous history of COVID-19 infection exhibited long COVID symptoms and fatigue was the most reported symptom (6). A study by Dryden et al. reported a prevalence of post COVID in South Africa at 82.1% among hospitalised patients one month after hospital discharge (7). Concerning the severity of COVID-19 infection, it was found that 60% of the patients with mild COVID-19 infection experienced more than one long COVID symptom and 35% of them experienced more than 3 long COVID symptoms for a period of two months (8). In addition, a quarter of the patients reported non-recovery from COVID-19 (8).

Interventions ranging from pharmaceutical or herbal supplements to physical rehabilitations have been implemented in various countries to address specific symptoms of long COVID (9). For instance, in Italy, a clinical trial that included olfactory rehabilitation along with supplements for a period of 30 days resulted in significant improvement in patients with anosmia/hyposmia (10). Another intervention in Spain offered personalised tele-physiotherapy for a duration of 20–30 min per session with a frequency of 3–5 times per week. The study showed that there was an improvement in the functional capacity of the patients and proved to be an effective intervention with an added advantage of home-based rehabilitation for long COVID symptoms (11). In LMICs, the burden of long COVID combined with the absence or the uneven distribution of rehabilitation services, and limited health infrastructure further exacerbates healthcare disparities and limits the access to essential care (12). Despite the high burden of long COVID in South Africa, there is lack of evidence on multidisciplinary interventions for long COVID in this setting (13). Clinical intervention such as dual antiplatelet therapy (DAPT) for the treatment of fibrin amyloid microclots to improve the platelet pathology post COVID-19 (14) are found in the literature, but patient-centred rehabilitation and support interventions focusing on symptom management are limited.

To fill this gap, we conducted a situational analysis and implemented group rehabilitation and self-management program in Johannesburg, South Africa (15). The implemented intervention was based on the HOPE digital peer-supported self-management intervention for long COVID in the UK (16). The concept of the intervention depended on two important themes of positive psychology—Hope and Gratitude (16). The duration of the intervention was 8 weeks and consisted of weekly in-person group sessions complimented by home-tasks and self-management exercises, with the option to adapt tasks to each person's abilities. The core of the sessions involved research staff facilitating discussion around participants' post-COVID-19 experiences, challenges and self-management strategies whilst encouraging group support and education on post-COVID-19 complications. Selected sessions invited rehabilitation specialists (e.g., psychologist, physiotherapist, dietician) for additional expertise (15). The main aim of the current study is to evaluate the acceptability and feasibility of this intervention.

## Materials and methods

This study employed a mixed methods design to assess the feasibility and acceptability of the group intervention for long COVID.

### Participants

Participants aged 18 years and over, self-reported or diagnosed with COVID-19 with at least one symptom lasting for more than two months, and willing to provide consent for the study were included in the intervention. The participant pool included both the patients as well as hospital staff who were affected by COVID-19. Participants with severe medical or psychiatric conditions affecting their ability to consent for the study and those requiring higher level of care were excluded from the study.

### Method of recruitment

The participants were recruited from the healthcare facility, specifically from the general, medical, and rehabilitation out-patient departments. Recruitment was done in-person by research staff, as well as through referrals from the healthcare workers. Further participants were recruited by snowball strategy based on patient or healthcare worker referral. The recruitment was from July to October 2023.

### Recruitment setting

The study was conducted within the Ekurhuleni District in Gauteng, South Africa. The study site was Tembisa Provincial Tertiary Hospital (Tembisa), a government-funded hospital catering to the general public.

### Sample size

The initial recruitment was planned up to 60 participants who meet the eligibility criteria into 6–7 groups. Due to intervention being a pilot, it was decided that all the participants would be receiving the intervention. A target sample size was deemed adequate for this study, informed by evidence that sample sizes of 24–50 were sufficient to estimate the key parameters of an efficacy and feasibility trial (17).

### Intervention

#### Description

The goal of the intervention was to improve the physical and psychological well-being of those affected by post-COVID-19 complications. The intervention consisted of both group sessions and home-based tasks. Participants were divided into seven groups, with six to ten participants per group. A baseline assessment was conducted prior to the intervention, and the post-intervention assessment was scheduled within two weeks after the 8-week intervention.

#### Development

The group sessions of the intervention were based on WHO brochure: *Support for rehabilitation: self-management after COVID-19-related illness* (18), as well as guidance from the intervention study conducted by Wright, H., et al. (16) on digital peer-supported self-management intervention co-designed by people with long COVID and the situation analysis (15). Each week had a specific theme to guide group facilitation and home tasks. The home tasks were designed based on recommendations from rehabilitation specialists as well as from the WHO self-management brochure (18). Home-tasks focused on self-management and self-moderation, breathing and physical exercises with the participants having the option to adapt the tasks according to their abilities.

#### Delivery

The group sessions took place on the Tembisa Hospital premises. The research staff facilitated discussion around participants' post-COVID experience, challenges, and management strategies. The sessions encouraged group support, education around long-COVID complications, self-management strategies and goal setting. The sessions and home material were mostly given in English, with some interaction in isiZulu or sePedi as required by participants. At selected weekly sessions, a rehabilitation specialist (physiotherapist, occupational therapist and/or psychologist) guided the group discussions. If participants were unable to attend the group session, they were given a summary of the session and an explanation of the weekly home-tasks telephonically by the team. The home-tasks were regularly monitored through the participants documentation and their subjective views of progress. To increase compliance, participants were reimbursed for their time and travel costs.

### Data collection

The acceptability and feasibility were determined using a 20-item questionnaire, consisting of both close-ended and open-ended questions applied at the end of the intervention. Questions 1–7 utilised Likert scale, ranging from 1 being “I don't agree at all” to 5 being “totally agree”, to assess the participants' overall perception of the intervention. The questionnaire also included questions related to breathing and physical exercises, materials used during the sessions, duration of the sessions, what the participant liked or disliked about the sessions and their recommendations. In addition to the questionnaire, semi-structured interviews using an interview guide with at least one participant per group were conducted, a total of nine interviews.

### Data analysis

We conducted the descriptive analysis in Excel spreadsheets. The socio-demographic variables were represented using frequency tables. The responses for acceptability and feasibility questionnaire were analysed and presented using frequency tables.

The interviews with the participants were recorded and transcribed in Microsoft Word by the research staff. Two researchers reviewed the transcripts for consistency and accuracy. The anonymity of the interviewers was safeguarded by using the assigned ID numbers. Since the themes for the interviews were pre-determined, a deductive thematic analysis was used. An online software Taguette was used to code the transcripts. A table with the pre-determined themes, and codes are attached in the Supplementary Material S1.

## Results

### Baseline characteristics of the participants

A total of 67 participants were initially recruited for the intervention. Of the 67 participants, five left the study after baseline evaluation, one missed endline visit due to work commitments, one attended only one week of intervention and then dropped out of the study, and one missed endline visit due to illness resulting in a total of 59 participants who completed the sessions and both pre- and post-intervention evaluation. Majority of the participants were aged less than or equal to 40 years (57.6%), females (74.6%), and single (69.5%). Fewer than 50% of the participants were formally employed. Most of participants had never received any rehabilitation for alleviating the symptoms (88.1%). Only 6.8% of the participants were smokers but 52.5% consumed alcohol. Table 1 shows the baseline characteristics of the participants.

**Table 1:** Baseline characteristics of the participants.

Variable	% (*n*/*N*)
Age	
Mean (SD): 39.7 years (12.7)	
</= 40 years	57.6% (34/59)
>40 years	42.4% (25/59)
Sex	
Male	25.4% (15/59)
Female	74.6% (44/59)
Marital status	
Single	69.5% (41/59)
Married/living with a partner	25.4% (15/59)
Divorced/separated	1.7% (1/59)
Widowed	3.4% (2/59)
Educational status	
Primary school	1.7% (1/59)
High school	33.9% (20/59)
Vocational training	33.9% (20/59)
University or higher	30.5% (18/59)
Employment status	
Self-employed	3.4% (2/59)
Formally employed	44.1% (26/59)
Unemployed	50.8% (30/59)
Retired	1.7% (1/59)
Industry employed	
Hospital staff^a^	71.2% (42/59)
Others	28.8% (17/59)
Did you have to leave work or reduce workload since the infection^b^	
Yes	47.5% (28/59)
No	32.2% (19/59)
Received rehabilitation	
Yes	11.9% (7/59)
No	88.1% (52/59)
Type of rehabilitation received	
Physiotherapy (chest, movement, strength)	6.8% (4/59)
Psychology/counselling/debriefing	0
Occupational therapy	0
Speech therapy	0
Dietician	0
Others	1.7% (1/59)
Currently smoke	
Yes	6.8% (4/59)
No	93.2% (55/59)
Currently consume alcohol	
Yes	52.5% (31/59)
No	47.5% (28/59)
Frequency of alcohol consumption	
Daily	0
Once or twice a week	19.4% (6/31)
Two or three times a month	25.8% (8/31)
Once a month or less	54.8% (17/31)
Never	0

^a^ Hospital staff were not just nurses, doctors and physiotherapists but also people working at the hospital such as finance, admin, cleaning staff etc.

^b^ Missing entries—12 participants didn't respond.

### Acceptability and feasibility of the intervention

#### General perception of the intervention

**Figure 1:**
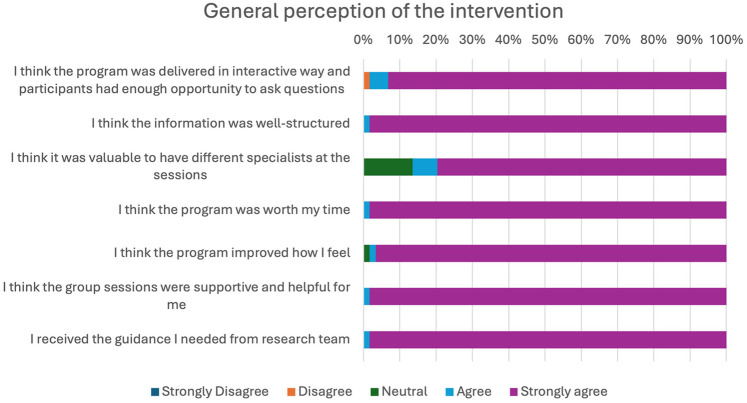
General perception of the intervention assessed through Likert scale.

A high proportion of the participants expressed a positive opinion about the intervention structure, information conveyed and its impact on their health. Nearly 98.3% of the participants stated that the group sessions were supportive as well as helpful for them and that they received enough guidance from the team members. Of the 59 participants, 58 attended more than half of the planned sessions emphasizing the participant's enthusiasm in attending the program, see Figure 1.

#### Delivery of the intervention

The perception about the delivery methods used in the intervention was gathered through an open-ended question in the questionnaire. All the participants had a positive response towards the delivery method of the intervention. The responses were coded the following: useful and helpful, satisfactory, interesting, productive, well-structured, progress monitored and provided guidance and encouragement.

One of the participants complimented that the proposed intervention was executed according to the plan.

“ … the design of the pilot intervention, to me, I can say it was very effective, it was very therapeutic. … everything was according to the way they explain what is going to happen throughout the whole process.” (Female, 41yrs).

Facilitators as well as the participants refrained from making judgements, thereby providing a space for open communication and interaction.

“No one was forcing anyone, no one was judgmental.” (Female, 30yrs)

#### Duration of the intervention

**Figure 2:**
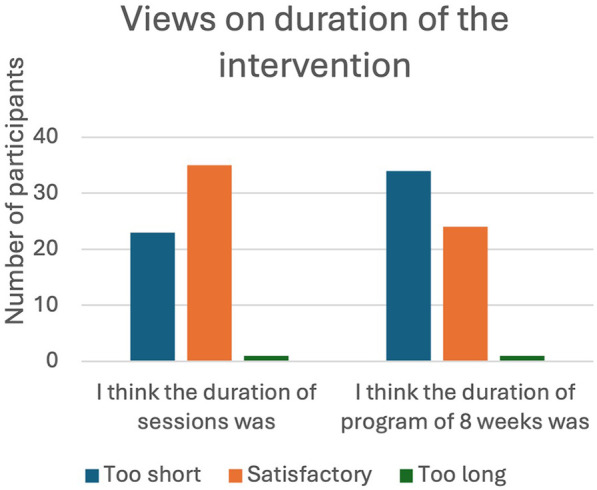
Views on duration of the intervention.

**Figure 3:**
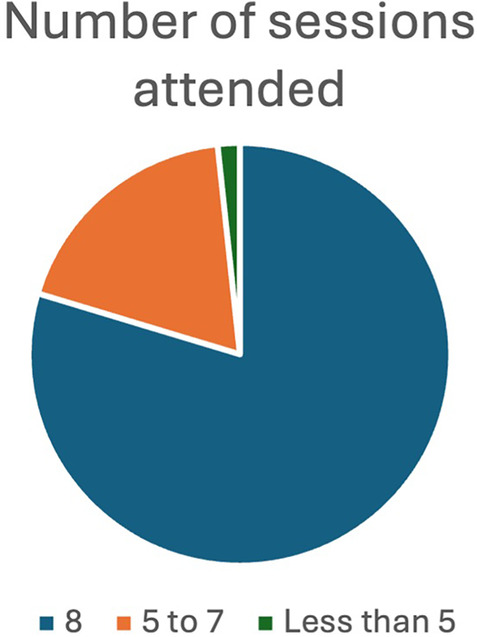
Number of sessions attended.

A total of 34 (57.6%) of the participants felt that the duration of the 8-week intervention was too short but the duration of each session, which took place for an hour, was satisfactory (59.3%). Almost 80% attended all the eight sessions and only one participant (1.7%) attended only two sessions (Figures 2, 3).

A similar standpoint on the duration of the intervention was reiterated during the interviews.

“I feel like it was very short. We needed more time, like, since some of us were going through a lot, and this was helping us a lot.” (Female, 32yrs)

Some participants mentioned that few of them were late which affected the duration of the sessions.

“ … it was very short and most of the people were coming late people don’t have time management, but our time was very short” (Female, 37yrs)

One of them noted that it took time for some of the participants to express their feelings, and by the time they were comfortable, the sessions had already concluded.

“I can say the duration was too short. Because there are some of the things that some people needed to like express or have a say for the first two weeks or three weeks, people are still shy to express their feelings and everything. And then when you get used to people trying to get to us, then session has ended. So, I think the duration should be extended” (Male, 25yrs)

#### Materials used for the intervention

In the questionnaire, majority of the participants (93.2%) claimed that the educational materials were interesting and 52.5% stated that they were personally relevant. None of them mentioned that the materials were either confusing or too long (Table 2).

**Table 2:** Opinion on the educational materials used in the intervention.

Statement	Answer	% (*n*/*N*)
I think the education material was	Interesting	93.2% (55/59)
	Credible	3.4% (2/59)
	Logical	20.3% (12/59)
	Comprehensible	37.3% (22/59)
	Personally relevant	52.5% (31/59)
	Confusing	0
	Complete	25.4% (15/59)
	Too long	0

During the interviews, the participants also stated that materials were useful and guided them during the home-based tasks. The participants also mentioned that the materials were helping them to recall a certain component or method, that they had forgotten.

“They are very useful. Even now I am still using them at home so the more I can get, the more I can use them at home. They are very useful because even if you forgot something you can refer to the material and check it is only medication that is giving me problem.” (Female, 58yrs)

However, one of the participants mentioned that some of the goals listed in the material were difficult to achieve and that it required more time and practice.

“I think … some of the goals is not easy to accomplish … I keep on using them [materials] to give myself time to accomplish some of the goals.” (Male, 25yrs)

#### Perceptions about the facilitators and external consultants

The facilitators played a major role in the implementation of the intervention. The participants recognised the empathetic nature of the facilitators and specialists and praised them for their work and involvement during the sessions.

“Well, we are grateful we've got the best facilitators in our facilities, very felt like it was. They understand what we come through. They were there for us from day one. Coming to exercising. It was tough, exercising, because we were not used to it. The first week was very tough. And then they had our hands they were there for us. They were always giving us courage to go on to continue always motivating us.” (Female, 32yrs)

One of the participants commended the emotional support rendered by dieticians and that they enjoyed the physical exercises.

“I enjoyed dietitian a lot because it was really helping to reducing fats in our bodies by balancing our diet and when it comes to physio, I enjoyed exercises.” (Female, 37yrs)

Another participant recounted their struggle with sleeping difficulty and noted that the advice from facilitators provided them necessary comfort and helped them overcome the issue.

“I have, you know, before I joined the program, I was struggling to sleep. I used to struggle a lot to sleep. And I used to experience a lot of headaches. Then when I engaged with the research team and the facilitator, then they you know, they provided me with the necessary information: how about you try this meditation? How about you try your physical walks, how about you change your diet. And since then, I'm sleeping like a baby, I'm enjoying life.” (Male, 31yrs)

The participant further mentioned that the facilitators were easily accessible and rendered support whenever possible.

#### Barriers or difficulties perceived by the participants

Infrastructure, time and language were the main barriers perceived by the participants which was captured during the qualitative interviews. Concerning infrastructure, absence of a permanent room with adequate space, and insufficient number of speakers for optimal acoustics were the main issues faced by the participants.

“We were using a laptop to listen, I felt like we needed a speaker. Because the volume was down. If it’s because the speakers were there that one was a good one, for meditation, and again from exercising session there was this one we didn't exercise because we didn’t have enough space, so I feel like in that one we needed to be outside not in the boardroom.” (Female, 32yrs)

“Given enough time and having a specific place that belonged to the group sessions without having to switch boardrooms.” (Female, 73yrs)

The sessions took place at different times depending on group availability, in general between 08:00–10:00 and 11:00–13:00. Some participants felt that the sessions could be organised a bit earlier. The heat and exhaustion from the travel made the participants tired before the start of the session.

“It was 12h00 and at the end some of us were coming late and is summertime is too hot so when we arrive here, we are already tired so if you can shift time to earlier hours maybe you can even extent time because the time was very short … .” (Female, 58yrs)

There were mixed feelings expressed regarding languages used. The sessions were conducted mainly in English with some isiZulu and sePedi. One participant mentioned that they weren't familiar with Zulu language and had difficulty understanding certain words during the session.

“To be honest with you I don’t know isiZulu and sePedi I am trying to combine all the languages … … some of the words that I cannot hear so when coming to language isiZulu is very difficult for me but other than that I don’t have a problem” (Female, 37yrs)

At the same time*,* two respondents claimed that the facilitators explained the contents in different local languages and in English, so that the information was well-conveyed and understood by all the participants.

“If someone does not understand English, you were able to translate it into their home language.” (Female, 73yrs)

#### Perceived benefits of the intervention

In the questionnaire, 27.1% (16/59) felt that the intervention provided them with a positive experience, 16.9% (10/59) claimed that the intervention provided guidance and education and 59.3% (35/59) stated that components of the sessions such as dietary advice (8/59), grief sessions (5/59), and physical and breathing exercises (21/59) as well as external visits from health care consultants (1/59) contributed to their well-being.

Participants renumerated the impacts of the intervention in the interviews, highlighting its positive influence on their health. The most common narrative emphasised an improvement in self-confidence and self-reliance. Long COVID symptoms had previously left them feeling helpless and hopeless. The intervention helped them gain both physical and mental strength, that significantly improved their quality of life.

“When one had COVID, the primary symptoms that I experienced, personally, was shortness of breath, so I couldn't breathe normally. So, when I became part of the program, I was taught on breathing exercises. And after participating in breathing programs, then I know how to I know of the different breathing techniques. So, it has actually enlightened me.” (Male, 31yrs)

“On my side, it was self-confidence, and I have managed to find myself because I am capable of doing some of the things, I did not have self-confidence so when I was attending these sessions, I have regained my self-confidence.” (Female, 58 yrs)

In addition to improving health, the intervention also helped create awareness and improve knowledge on long COVID symptoms and overall health.

“These sessions helped me a lot, since it was something that I was not expecting. I have learned a lot. Never mind that I had covid. I have learned that I can cope from anything, and I can continue to train and exercise.” (Female, 73 yrs)

“The intervention for me came in very handy if I can use the term because I think one had a lack of knowledge on certain aspects of health and overall well-being. So, it helped with, with coping mechanisms, we were taught, or we were taught on programs that actually assist in terms of the lifestyle, and also the general well-being of an individual.” (Male, 31yrs)

“ … the whole, the whole group was fine. It was productive. And like we learned a lot. We learned how to open up learned about the physical exercises, meditation, how to cope with stress, so it was helpful a lot.” (Female, 30yrs)

#### Participants' recommendations

The first recommendation was to expand the intervention to other locations such as schools, churches, and hospitals, so that others who have been affected by long COVID can benefit from the rehabilitation.

“If you can go into hospitals, if you can go into the communities. If you can go to churches, if you can go to any relevant institution to try and impact or provide this information that we've received.” (Male, 31yrs)

Concerning infrastructure, one of the respondents suggested the need for more space, especially for physical exercises. Few of the respondents had recommended some changes in the materials used for the intervention. One said that there needs to be visuals on the pamphlets, benefitting people who can't read or write the language.

“I can say maybe sometimes for some people, maybe it’s hard for them to like read, for example, maybe the pamphlets that you've given us the home test and everything, maybe it’s harder for them to read, if maybe there can be a possibility of visuals.” (Male, 25yrs)

Another suggested that the content must also include sensitive topics such as sexual well-being.

“I think, I can say some people. Sometimes they need a psychologist whereby some of the people are afraid to talk maybe about their sexual tension. I think those things some people are not, like free to talk about them, but are some things that affect daily life, our daily lives. So, I think those deep things, you must be able to access and motivate people more.” (Male, 25yrs)

The most common suggestion was to increase the duration of the intervention. The participants felt that a long-term program can immensely help them manage their symptoms and provide them with a support system.

“I can say the duration was too short. Because they are some of the things that some people were needed to like express or say for the first two weeks or three weeks, people are still shy to express their feelings and everything. And then when you get used to people trying to get to us to people, then session has ended. So, I think the duration should be extended” (Male, 25yrs)

Final recommendation was a request to follow-up after six months from the team for a wellness check and to receive feedback from the participants.

“I think because we are many, once in a while, maybe after six months you can call us together to share our experiences at home and to check us if we are still doing good … ” (Female, 58yrs)

## Discussion

The study demonstrated that the group intervention for managing long COVID symptoms was both acceptable and feasible in the South African context. Participants reported a range of positive impacts and offered constructive suggestions for further improvement and scale-up. Dropouts were primarily attributed to personal factors—such as employment demands, physical illness, or travel constraints—rather than group-related issues like discomfort with group dynamics or disinterest in intervention content.

The current study emphasized the importance of rehabilitation after COVID-19. Patients observed improvements in well-being, for instance sleep and physical capacity, and in self-confidence. Similar outcomes were seen in Berentschot et al's study, wherein significant improvements were recorded post rehabilitation for COVID-19 hospitalised patients compared to those without rehabilitation (19). A systematic review of long COVID interventions had also found that a combination of physical and mental health rehabilitation accelerates patient's recovery and enhances the quality-of-life indices (20).

One of the major highlights of the implemented intervention was the format of group sessions. A systematic review on facilitated group work among patients with chronic conditions has demonstrated statistically significant improvements in patients' health outcomes (21). Specific symptoms such as pain and fatigue seem to improve after rehabilitation (21). Albeit the effect being not long-standing, the study highlighted the need for integration of rehabilitation with standard care routine (21). In the present study, 98.3% agreed that the group sessions were supportive and helpful, highlighting that the group sessions were a success. However, some of the participants mentioned that few were shy and initially hesitated to share during the group sessions, especially during the grief session. A study that evaluated a group empowerment and training session for diabetes management in South Africa also stated that the some of the patients were reluctant and not motivated to engage in group activities (22). Research states that factors such as social anxiety, fear of shame and humiliation by the peers, and fear of instigating anger within peers could be the reason for hesitation to disclose personal experiences during group sessions (23).

Participants expressed appreciation for the facilitators of the intervention, specifically commending the professionalism, approachability, and competencies of the research staff. A qualitative study on stakeholder's perspective of South Africa's rehabilitation infrastructure found that facilitator's professional expertise had an impact on the quality of rehabilitation, which in turn affected patient's health outcomes (21). Similar opinion regarding the skillset of the facilitators were obtained in Selohilwe et al's study (24). Lack of judgement from peers as well as the facilitators in the present study helped the participants to come forward and share their health distress openly. This could be attributed as one of the success factors of the intervention.

Maart et al's study (25) found that financial constrains present in the public healthcare system led to inadequate availability of infrastructure and assistive devices for rehabilitation. Another study which investigated the challenges and opportunities for implementing task-sharing counselling intervention for depression at the primary healthcare setting in South Africa also highlighted infrastructure challenges, particularly, lack of physical space (24). This barrier was reemphasized by the participants in the current study. The participants stated that there was a lack of adequate infrastructure such as permanent room for the sessions and enough space for physical exercise, which impacted the delivery of the intervention. Maart et al's study (25) also pointed out the lack of transportation for patients to access rehabilitation services due to shortage of healthcare facilities. To negate this, the study participants were incentivized through reimbursement of their transportation costs, which motivated them to participate in the intervention. Similar incentives must be incorporated in the healthcare system to attract patients who need rehabilitation, alternatively, embedding these interventions within communities may make them more accessible.

### Strengths of the study

The long COVID group intervention implemented in Johannesburg, South Africa was the first of its kind. The aftereffects of COVID-19 in Africa have been underestimated and absence of rehabilitation along with standard post-COVID-19 care might affect the quality of life of patients. The implemented intervention not only was perceived as effective but was feasible as well as acceptable by the participants. The current rehabilitation program, which focused not only on patients but also on hospital staff who were affected by COVID-19, was another key highlight of the study. The consistent attendance of participants reflected their enthusiasm and commitment to the program.

### Study limitations

The intervention was conducted over a limited period of eight weeks, during which participants expressed a preference for a longer program with appropriate follow-up sessions. The intervention's location emerged as another key limitation, with participants noting that community-based delivery would have facilitated easier access. Interviews with selected participants were conducted in the regional language (isiZulu) and subsequently translated into English for analysis; however, nuances or culturally specific expressions may have been lost in translation.

## Conclusion

Rehabilitation post-COVID has a potential to improve quality of life, and this study shows that a focused intervention for supportive care was well-accepted by the participants. The analysis showed good feasibility factors such as a low loss-to-follow-up rate and high overall session attendance. The interviews, along with the quantitative questionnaire, highlighted a positive perception and impact of the intervention, emphasizing its acceptability. Similar long standing rehabilitation programs should be considered as part of the standard care package for chronic respiratory illnesses and long COVID.

## Data Availability

The qualitative data generated and analyzed in the current study are not publicly available. The data available from the corresponding author on reasonable request.
