# Supplementary material for: Acceptability and feasibility of a group intervention for long COVID in Johannesburg, South Africa: a mixed-method study
Source: Front Health Serv. 2025 Oct 30;5:1666387. doi: 10.3389/frhs.2025.1666387 (PMC12611963; doi:10.3389/frhs.2025.1666387)
Supplement: Supplementary file 1 [file Datasheet1.docx]

**Supplement 1**

Acceptability and feasibility of the intervention – Qualitative results (Codes and sub-codes)

| **Themes** | **Codes** | **Sub-Codes** |
| --- | --- | --- |
| Barriers | Barriers_Infrastructure | Barriers_Infrastructure_ Speakers |
|  |  | Barriers_Infrastructure_Absence of a permanent space |
|  | Barriers_Time |  |
|  | Barriers_Language |  |
|  | Barriers_Transport |  |
| Benefits | Benefit_Interpersonal | Benefit_Interpersonal_Language |
|  |  | Benefit_Interpersonal_Creating awareness |
|  |  | Benefit_Interpersonal_No Judgement |
|  | Benefit_Intrapersonal | Benefit_Intrapersonal_Self-realisation |
|  |  | Benefit_Intrapersonal_Confidence building |
| Design of the intervention | Design of the intervention | Design of the intervention_Effective |
|  |  | Design of the intervention_Relevant |
|  |  | Design of the intervention _Feedback |
| Duration of the session | Duration of the session | Duration of the session_More frequent |
|  |  | Duration of the session_Short |
|  |  | Duration of the session_Importance of time |
| Facilitators | Facilitators_Expertise | Facilitators_Expertise_Emotional support |
|  |  | Facilitators_Expertise_Empathetic |
|  |  | Facilitators_Expertise_Specialists |
| Materials used in the intervention | Materials_Achievable goals |  |
|  | Materials_Guide |  |
| Recommendations | Recommendations_Materials | Recommendations_Materials_Visual graphics in the Pamphlets |
|  | Recommendations_Time | Recommendations_Time_Longer duration of the intervention |
|  | Recommendations_Design | Recommendations_Design_ Feedback and follow-up |
|  | Recommendations_Intervention | Recommendations_Intervention_Settings |
